# Supplementary material for: Dual Control of LDL-cholesterol Levels by ANGPTL3 and ANGPTL8
Source: bioRxiv. 2026 Oct 28:2026.03.30.715445. Preprint. [Version 2] doi: 10.64898/2026.03.30.715445 (PMC13733725; doi:10.64898/2026.03.30.715445)
Supplement: Supplementary file 1 [file media-1.pdf]

Supplementary Materials for

Dual Control of LDL-cholesterol Levels by ANGPTL3 and ANGPTL8

Yanqing Xu PhD[2], Fei Luo MD, PhD[2], Justin Fletcher PhD[3], Melissa Inigo PhD[3], Shawn Burgess PhD[3], Guosheng Liang PhD[2,3], Lisa N. Kinch PhD[1,4], Julia Kozlitina, PhD[5], Jonathan C. Cohen PhD*[3], Helen H. Hobbs, MD*[1,2]

[1] Howard Hughes Medical Institute, University of Texas Southwestern Medical Center, Dallas, TX 75390

[2] Department of Molecular Genetics, University of Texas Southwestern Medical Center, Dallas, TX 75390

[3] Center for Human Nutrition, University of Texas Southwestern Medical Center, Dallas, TX 75390

[4] Department of Molecular Biology, University of Texas Southwestern Medical Center, Dallas, TX 75390

[5] Eugene McDermott Center for Human Growth and Development, University of Texas Southwestern Medical Center, Dallas, TX 75390

Short title: ANGPTL3 and ANGPTL8 in Cholesterol Metabolism

***Corresponding author:** Helen Hobbs: Helen.Hobbs@UTSouthwestern.edu

***Co-corresponding author:** Jonanthan Cohen: Jonathan.Cohen@UTSouthwestern.edu

University of Texas Southwestern Medical Center (UTSW)

Dallas, TX 75390-9046

Expanded Materials and Methods:

- Supplementary methods
- Supplementary table 1-6
- Supplementary figure 1-7

1 **Supplementary Methods**

2 **Table1. Materials: Reagents used in the experiments described in this paper.**

REAGENT	SOURCE	IDENTIFIER
**Antibodies (Ab)**		
Donkey Anti-goat IgG 2º Antibody (Ab)	Jackson ImmunoResearch	705-035-003
Donkey Anti-mouse IgG 2º Ab	Jackson ImmunoResearch	715-035-150
Goat Anti-mouse IgG, Fcy fragment-specific	Jackson ImmunoResearch	115-035-071
Goat Anti-rabbit IgG 2º Ab	Jackson ImmunoResearch	111-035-144
Goat Anti-ANGPTL3 (A3) polyclonal (p)Ab	Bio-Techne	AF136
Mouse Anti-ANGPTL8 (A8) monoclonal (m)Ab	In-house, as described[53]	19H4
Mouse Anti-V5 mAb	Invitrogen	46-1157
Rabbit Anti-A3 mAb	In-house (see below)	2G5
Rabbit Anti-ApoA5 pAb	US Biological	139307
Rabbit Anti-ApoB pAb	Abcam	Ab20737
Rabbit Anti-ApoE mAb	Abcam	Ab183596
Rabbit Anti-Calnexin pAb	Enzo	ADI-SPA-860
Rabbit Anti-Flag pAb	Millipore Sigma	F7425
Rabbit Anti-GFP mAb	Cell Signaling	2956S
**Bacterial Strains**		
E. coli BL21 (DE3) Competent Cells	Thermo Fisher Scientific	EC0114
**Buffers**		
Dulbecco′s Phosphate Buffered Saline (DPBS)	Millipore Sigma	D8537
Laemmli SDS Sample Buffer, Reducing 6x	Thermo Fisher Scientific	J61337.AC
Laemmli SDS Sample Buffer, Non-reducing 6x	Thermo Fisher Scientific	J60660.AC

NativePAGE 20x Cathode Buffer Addictive	Invitrogen	BN2002
NativePAGE™ Sample Prep Kit	Invitrogen	BN2008
NativePAGE 20x Running Buffer	Invitrogen	BN2001
Phosphate Buffered Saline (PBST)	Millipore Sigma	P3563
Phosphate Buffered Saline (10x PBS)	Millipore Sigma	6505-4L
Sodium Chloride Irrigation (0.9%), USP	Baxter	2F7123
Trans-Blot Turbo 5x Transfer Buffer	Bio-Rad	10026938
UltraPure™ 0.5M EDTA, pH 8.0	Thermo Fisher Scientific	15575038
**Cell Lines**		
QBI-HEK293A	Obiogene, formerly Quantum Biotechnologies	QBI-293A-2
**Chemicals**		
Acetic Acid	JT Baker	9515-03
Bovine Serum Albumin (BSA)	Sigma	A3803
FuGENE® 6 Transfection Reagent	Promega	E2692
Glycerol	Millipore Sigma	G9012
Heparin Sodium Salt	Sigma	H3393-500KU
Imidazole	Sigma-Aldrich	56750
Isopropyl β- d-1-Thiogalactopyranoside (IPTG)	Millipore Sigma	I6758
Kanamycin Sulfate	Millipore Sigma	K1377
NativeMark™ Unstained Protein Standard	Thermo Fisher	LC0725
Ni-NTA Agarose	Qiagen	30230
Potassium Bromide (KBr)	Sigma	P0838
Protease Inhibitor Cocktail (cOmplete™ Mini EDTA-free)	Millipore Sigma	11836170001

Sodium Hydrochloride (NaCl)	Sigma-Aldrich	S3014
Tris Hydrochloride (Tris HCl)	Sigma-Aldrich	10812846001
cis-4,7,10,13,16,19-Docosahexaenoic Acid	Sigma	D2534-1G
Linoleic Acid	Sigma	L1376-10G
γ-Linolenic Acid	Sigma	L2378-1G
Oleic Acid	Sigma	O1008-5G
Palmitoleic Acid	Sigma	P9417-1G
Palmitic Acid	Sigma	P5585-10G
Stearic Acid	Sigma	S4715-1G
**Kits**		
Cholesterol Quantitation Kit	Sigma Aldrich	MAK043
EnzChek™ Phospholipase A1 (PLA1) Assay Kit	Invitrogen	E10219
Gel Filtration HMW Calibration Kit	Cytiva	28403842
High Sensitivity Triglyceride (TG) Assay Kit	Sigma Aldrich	MAK264
Human A3 ELISA kit	Bio-Techne	DANL30
Lipoprotein Lipase (LPL) Activity Assay	Cell Biolabs	STA-610
Mouse ApoB-100 ELISA Kit	Abcam	ab230932
Mouse A3 ELISA Kit	Antibodies online	ABIN1884517
Phospholipid Quantification Assay Kit	Millipore Sigma	CS0001
Phospholipids C Reagent	Wako life Sciences	997-01801
Pierce BCA Protein Assay Kit	Thermo Fisher Scientific	23224
Power SYBR Green PCR Master Mix	Applied Biosystems	4368708
Total Cholesterol Reagents	Thermo Fisher	TR13421
Triglycerides Reagent	Thermo Fisher	TR22421
TaqMan Reverse Transcription Reagents	Applied Biosystems	N8080234

RNeasy Plus Universal Mini Kit	Qiagen	73404
V5-tagged Protein Purification Kit Ver.2	Cosmo Bio	MBL-3317
**Medium**		
Dulbecco′s Modified Eagle Medium (DMEM) with L-Glutamine, 4.5g/L Glucose	Corning	10-0130-CV
Fetal Bovine Serum (FBS)	Corning	35-015-CV
Luria Broth (LB)	Fisher Scientific	BP1426-500
Opti-MEM™ Reduced Serum Medium	Gibco	11058-021
**Oligonucleotides**		
See SI Appendix, Table S1		
**Recombinant DNA**		
pcDNA3.1	Lab	
pET28a	Novagen	69864
**Recombinant Proteins**		
Human-A3-V5-6xHis	Lab	
Human-A8-V5-6xHis	Lab	
Human-EL-Myc-Flag	Lab	
Mouse-A3 (aa17-224)-6xHis	Lab	
**Software and Algorithms**		
Image Studio Lite v5.2	LI-COR	Version 5.2.5
GraphPad Prism 10	GraphPad	Version 10.0.0

SnapGene	SnapGene	Version 6.0.2
**Virus**		
AAV8-TBG-GFP	Vector Biolabs	QB63528
AAV8-TBG-mA8-Flag	Vector Biolabs	QB63094
AAV8-TBG-mA8 (mutant)-Flag	Vector Biolabs	Q-0163-1
**Other**		
Amicon Ultra Centrifugal Filter (10 kDa MWCO)	Millipore Sigma	UFC8010
AKTA Pure Chromatography System	Cytiva	29018224
Chow Diet (rodent) 16% Protein	Teklad Global	2016
4–15% Criterion™ TGX™ Precast Midi Protein 26-well Gel	Bio-Rad	5671085
4–15% Criterion™ TGX™ Precast Midi Protein 18-well Gel	Bio-Rad	5671084
4–15% Criterion™ TGX™ Precast Midi Protein Gel (Prep+2 well)	Bio-Rad	5671082
HisTrap HP, 5 ml	Cytiva	17524802
Immobilon®-PSQ PVDF 0.2 µm Membrane	Millipore Sigma	ISEQ00010
Instant Nonfat Dry Milk	Nestle	
Millex-HV Syringe Filter Unit (0.45 µm, PVDF membrane)	Millipore Sigma	SLHV033RS
MP Biomedicals™ Fat Free Diet, Pellet (high sucrose diet)	Thermo Fisher Scientific	ICN90168310
NanoDrop™ 8000 Spectrophotometer	Thermo Fisher Scientific	ND-8000-GL
NativePAGE™ Bis-Tris Mini Protein Gels (3-12%) 1.0 mm	Thermo Fisher Scientific	BN2011BX10
Open-Top Ultra-Clear Tube (13.2 mL)	Backman	344059
Superose 6 Increase 10/300 GL Column	Cytiva	29091596

Superdex 200 Increase 10/300 GL Column	Cytiva	28990944
SuperSignal™ West Pico PLUS Chemiluminescent Substrate	Thermo Fisher Scientific	34577
Trans-Blot Turbo RTA Midi 0.2 µm Nitrocellulose Transfer Kit	Bio-Rad	1704271

Mice

Mice were housed in a controlled environment (12-h light/12-h dark cycle) at ambient temperature of 23 ± 1°C, 60-70% humidity). For fasting/refeeding experiments, mice were entrained for at least 3 days. The mice were fasted overnight (5:00 PM-9:00 AM) and fed during the day (9:00 AM-5:00 PM). Experiments were performed at 9:00 AM (fasted) or 1:00 PM (4 h after refeeding).

A3$^{-/-}$ mice were generated by replacing the gene with a LacZ-Hyg cassette using homologous recombination. The mice were backcrossed with C57BL/6J mice as described.[38] *Ldlr*$^{-/-}$;*A3*$^{FL/FL}$ mice were generated as described in the legend to Figure 2. For *Ldlr*$^{-/-}$;*A3*$^{Nter/Nter}$ mice, a stop codon followed by a polyadenylation (polyA) tail was inserted at residue 225. F_0 founders were crossed with *Ldlr*$^{-/-}$ mice to generate heterozygous offspring (F_1), which were intercrossed to generate mice homozygous for the mutation. *Ldlr*$^{-/-}$;*A3*$^{FL/-}$ and *Ldlr*$^{-/-}$;*A3*$^{Nter/-}$ mice were generated by crossing *Ldlr*$^{-/-}$;*A3*$^{FL/FL}$ mice and *Ldlr*$^{-/-}$;*A3*$^{Nter/Nter}$ with *Ldlr*$^{-/-}$;*A3*$^{-/-}$ mice, respectively. *Ldlr*$^{-/-}$;*A3*$^{FL/Nter}$ heterozygous mice were obtained by intercrossing *Ldlr*$^{-/-}$;*A3*$^{FL/-}$ and *Ldlr*$^{-/-}$;*A3*$^{Nter/-}$ mice. Triple knockout *Ldlr*$^{-/-}$;*A8*$^{-/-}$;*Lipg*$^{-/-}$ mice were produced by crossing *Ldlr*$^{-/-}$;*A8*$^{-/-}$ mice with *Ldlr*$^{-/-}$;*Lipg*$^{-/-}$ mice following by intercrossing the double- heterozygous offspring. Similarly, *Ldlr*$^{-/-}$;*A3*$^{-/-}$;*A8*$^{-/-}$ mice were generated by crossing *Ldlr*$^{-/-}$;*A8*$^{-/-}$ mice with *Ldlr*$^{-/-}$;*A3*$^{-/-}$ mice. *Ldlr*$^{-/-}$;*A3*$^{FL/FL}$ and *Ldlr*$^{-/-}$;*A3*$^{Nte/Nter}$ mice were crossed with *Ldlr*$^{-/-}$;*A8*$^{-/-}$ mice. The double-heterozygous offspring were then intercrossed to generate *Ldlr*$^{-/-}$;*A3*$^{FL/FL}$;*A8*$^{-/-}$ and *Ldlr*$^{-/-}$;*A3*$^{Nter/Nter}$;*A8*$^{-/-}$ mice.

Genomic DNA was extracted from mouse tails and analyzed by PCR for genotyping. Primer sequences are listed in Table S1.

For the mouse experiments, no formal *a priori* sample sizes were calculated. Sample sizes were based on prior experience with similar mouse studies, the expected effect sizes, and the availability of the required genotypes. The number of animals used in each experiment is indicated in figure legends.

For experiments comparing genotype-defined groups, randomization was not used. For intervention experiments within the same genotype (Figure 3E, 5F and 6D), animals from the same cage/litter were randomly distributed between groups to minimize allocation bias. Experimental procedures were performed in a balanced order to minimize potential bias.

Liver Perfusion and VLDL Isolation

Livers from ad libitum fed mice were perfused exactly as described.[54] Mice were anesthetized with ketamine/xylazine (4U/g body weight) followed by an intraperitoneal injection of heparin (100 U). Perfusions were conducted for 60 min in a nonrecirculating mode using oxygenated buffer containing free fatty acids (FFA, 0.8 mmol/L) bound to albumin. Following each perfusion, VLDL-enriched fractions were isolated from perfusates by density gradient ultracentrifugation (d=1.006 g/mL using KBr). For heparin-treated samples, VLDL was isolated as described previously.[55] For non-heparin treated samples, perfusates (12 mL) were subjected to centrifugation (40,000 rpm in TH641 swinging bucket rotor, 18 h at 4°C) in ultra-clear open-top tubes. The top 1 mL was collected, and proteinase inhibitors were added before the samples were stored at -80°C.

Immunoblotting of VLDL proteins was performed as described.[55] VLDL-lipids (PC, cholesterol, TG) were quantified fluorometrically using kits (see Table 1).[55]

Protein Expression and Purification for Antibody Generation

A cDNA encoding mouse A3 (residues 17-224) was cloned into pET28(+) and the sequence was verified prior to the plasmid being introduced into *E. coli* BL21 cells. Kanamycin-resistant colonies were expanded in LB-kanamycin (1 L) to an OD_{600}=0.8, and then IPTG (1 mM) was added to induce expression, and the cells were grown for 3 h at 37°C.

Cells were pelleted (3325*g* for 30 min at 4°C), resuspended in 10 mL of buffer A (20 mM Tris HCl, 500 mM NaCl, 5 mM imidazole, 10% glycerol, pH 8.0) and lysed by sonication (3 sec on, 4 sec off, 5 min total) on ice. The lysates were clarified by centrifugation (15,000 rpm, 30 min, 4°C). The His-tagged proteins were purified using Ni-NTA resin (1 mL/L). Cell lysates were incubated with Ni-NTA resin equilibrated in buffer A at 4°C for 1 h on a shaker (200 rpm), washed 3x with buffer B (20 mM Tris HCl, 500 mM NaCl, 25 mM imidazole, 10% glycerol, pH 8.0), then eluted in buffer C (20 mM Tris HCl, 300 mM NaCl, 300 mM imidazole, 5% glycerol, pH 8.0). Purified A3-Nter (aa 17-224) was concentrated using a membrane filter (10-kDa) and then quantified by absorbance at A280, aliquoted (1mg/mL), snap-frozen, and stored at -80°C for antibody generation.

Immunoassays

Immunoblotting was performed after protein separation by SDS-PAGE (4-15%) as described previously.[52] Mouse plasma was diluted 1:5 in 0.9% NaCl and heated at 95°C for 5 min in Laemmli reducing sample buffer before electrophoresis. A total of 10 μL of diluted plasma was loaded per lane. To detect A3, plasma samples were prepared in non-reduced Laemmli buffer, and 5 μL was loaded per lane unless indicated otherwise in figure legends.

Membranes were incubated overnight at 4°C with the indicated primary antibodies (1:1000), except for the anti-A8 mAb (19H4, 0.6 mg/mL), which was used at a dilution of 1:250. The 2° antibodies (1:5000 dilution) were incubated with the membranes for 1 h at room temperature. A complete list of antibodies used for immunoblotting is provided in Table 1.

Native protein complexes were separated on NativePAGE (3-12%) Bis-Tris gels according to the manufacturer's instructions. Plasma from male mice (4-5/per group, 8-12 wks, refed for 4 h) was fractionated by Superdex 200 size-exclusion chromatography. Fractions containing immunoreactive A3 were pooled, concentrated by centrifugation using 10 kDa cut-off filters (5000*g* for 20 min at 4°C), and prepared in 1x NativePAGE sample buffer before electrophoresis. A total of 30 µL was loaded per lane, except for the sample from mice expressing A3-FL, for which 10 µL was loaded because of higher protein abundance.

Fractionated plasma was separated by native PAGE (first dimension) at room temperature for 1.5 h at 150 V using prechilled light blue cathode buffer in the upper chamber and 1× NativePAGE anode buffer in the lower chamber. Proteins were transferred to 0.2 µm PVDF membranes using 1× Turbo blot Transfer Buffer, washed with 8% acetic acid for 15 min, blocked in 5% nonfat milk for 1 h, and incubated overnight at 4°C with anti-A3 mAb (2G5). Lane strips were excised from the nondenaturing gel and subjected to SDS-PAGE under denaturing conditions in a second dimension. Proteins were then transferred to 0.22-µm nitrocellulose membranes and immunoblotted with antibodies against A3 (2G5) and A8 (19H4).

Immunoblots were imaged using an Odyssey FC Imager (LI-COR) and the band intensities were quantified with Image Studio Lite Ver 5.2 (LI-COR).

Quantitative Real-Time PCR

Total RNA was extracted from liver samples using an RNA extraction kit according to the manufacturer's instructions. RNA concentration and purity were determined using a NanoDrop spectrophotometer, and RNA integrity was assessed by agarose gel electrophoresis. First-strand cDNAs were synthesized from 2 μg total RNA using random hexamer primers and reverse transcription reagents (see Table 1).

Real-time PCR was performed using an Applied Biosystems real-time PCR system with SYBR Green (Table 1). Each reaction was carried out in a 20 μL volume containing 20 ng cDNA, 167 nM primers, and 10 μL SYBR Green PCR Master Mix, in duplicate.[56] Glyceraldehyde-3-phosphate dehydrogenase (*Gapdh*) mRNA and 18S ribosomal RNA served as the internal normalization control.

Adeno-Associated Virus Infection of Mice

Adeno-associated virus serotype 8 (AAV8) vectors expressing either GFP (control), mouse A8 or mutant mouse A8 under the control of the TBG promoter were administered by tail-vein injection at a dose of 1×10^{11} genome copies in 200 μL saline per mouse using a 1-mL syringe with a 30G×½" needle. After injection, mice underwent at least 3 fasting/refeeding cycles and were killed after the final fasting period.

Lipid Analysis and Lipoprotein Profiling

Hepatic lipids were extracted from liver (~50 mg) using Folch solution.[57] Levels of hepatic and circulating TG, cholesterol and PC were measured enzymatically as described.[22] Hepatic lipid levels were normalized to wet tissue weight (mg/g).

Plasma lipoprotein particles were size-fractionated by fast performance liquid chromatography using a Superose 6 Increase 10/300 GL column. Cholesterol and TG content of each fraction was measured enzymatically as described.[22]

LPL and EL Activity Assays

Heparin (1 U/g) was injected intravenously after 4 h of refeeding. Plasma was collected 15 min post-injection, isolated by centrifugation (2000*g* for 10 min at 4°C), and snap-frozen as described previously.[38] Total lipase activity and PLA1 activity in plasma were measured using enzymatic kits (Table 1) according to the manufacturers' instructions.[30,52]

To separate hepatic lipase (HL) and lipoprotein lipase (LPL), post-heparin plasma (250 µL/group) was fractionated on a 1 mL heparin-affinity column as described.[41,52] TG lipase activity in each fraction (20 µL/fraction) was measured using a TG lipase activity kit according to manufacturer's instructions. Additionally, plasma (350 µL/group) was fractionated on a Superdex 200 size exclusion column, and odd fractions (10 µL/fraction) were analyzed for both TG lipase and PLA1 activities as described.[30,52]

Cell Lines and Transfections

QBI-HEK293A cells were grown in 10-cm dishes in complete medium [high sucrose DMEM plus 5% FBS] at 37 °C in 8.8% CO_2. Cells were transiently transfected with cDNAs encoding human A3, A8, or EL using FuGENE 6 according to the manufacturer's instructions. Conditioned medium was collected 72 h post-transfection, filtered through a 0.45 µm membrane to remove cell debris, and concentrated using a 10-kDa membrane filter (5,000*g* for 15 min at 4°C). The concentrated medium was used immediately or snap-frozen and stored at -80°C.

Enzyme-Linked Immunosorbent Assay (ELISA)

Plasma A3 was measured using a sandwich ELISA according to the manufacturer's instructions. In this assay, the capture and detection Abs both recognize epitopes in A3-Nter (residues 17-165). ApoB-100 levels in VLDL fractions isolated from liver perfusates were measured using a commercial ELISA kit which specifically detects the C-terminal region of ApoB according to the manufacturer's instructions. Conditioned medium containing V5-tagged A3, either alone or co-expressed with A8, was immunoprecipitated with an anti-V5 antibody, and the precipitated proteins were quantified using a human A3 ELISA kit according to the manufacturer's instructions. The linearity of each assay was confirmed by serial dilution of the samples.

Quantification of Circulating A3 by Selected Reaction Monitoring (SRM) Mass Spectrometry (MS)

To improve detection of low-abundance plasma proteins, we first removed albumin from the samples using an albumin depletion kit. The samples were digested with trypsin and spiked in with 2 pmol of each heavy-isotope labeled peptide: SFVEQQDNSI**R** (aa166-167); TGIQEPSENSLSS**K** (aa206-219). Lys was labelled by $^{13}C_6$, $^{15}N_2$, and Arg was labeled by $^{13}C_6$, $^{15}N_4$. Peptides were selected based on their specificity and digestion efficiency. All peptides were synthesized by Life Technologies Corporation. Mass spectrometry was conducted in UTSW Proteomic Core.

EL Inactivation by $A3_3$ and $A3_2A8$

Structural models of $A3_2A8$ and $A3_3$ bound to EL or LPL were generated using the AlphaFold Server[58,59] using sequences corresponding to the secreted forms of human proteins: A3-FL

(residues 17-460), A3-Nter (residues 17-221), A8 (residues 22-198), EL (residues 21-500) and LPL (residues 28-475). AlphaFold confidence metrics (ipTM/pTM) were used to assess model quality at the complex level. Because ipTM reflects confidence averaged across entire chains and the lipase-ANGPTL interaction interface is relatively small, we also generated "trimmed" models lacking low-confidence or disordered regions, EL (residues 46-485), A3 (35-152), and A8 (17-143). We refer to inhibitor-complex structures lacking disordered regions as "hinge" models because they are trimmed at a natural break, or hinge, in the N-terminal helix, allowing the remaining coiled-coil segment to bend at multiple angles (Table S5). Consistent with prior observations[59,60], the trimmed hinge models yielded the highest confidence metrics when complexed with EL, however, higher ipTM scores do not necessarily reflect confidence in the interaction interface itself. To better assess the EL interaction in both $A3_3$ and $A3_2A8$ complexes, we therefore focused on local interface quality, as reflected by AlphaFold3 inter-residue contact probabilities. Inter-residue contacts were defined as residue pairs with nonzero contact probabilities between EL and the primary interacting helix of the inhibitor complex, specifically, the A8 helix in the A3-FL_2A8 or the corresponding residues in the A3 helix (for the A3-FL_2A3-Nter complex, see heatmaps in Figure 6C), which more directly reflects the predicted strength and specificity of $A3_3$ and $A3_2A8$ binding surfaces. The sequence ranges of $A3_3$ and $A3_2A8$ with EL and LPL and their TM scores are provided in Table S5.

Table S1. Oligonucleotides Sequences Used for Genotyping and Sequencing.

**Genotyping:**	
A3$^{FL/FL}$	F-5’-TCTGCACATGCTCAGCAAGCACTGTGCCCC-3’
	R-5’-AGACAACAAGCCAGGGTCCAATTAAATTAAC-3’
A3$^{Nter/Nter}$	F-5’-TCTGCACATGCTCAGCAAGCACTGTGCCCC-3’
	R-5'- TGGCAACTAGAAGGCACAGTCGAGG-3’
A3$^{-/-}$	lacZ-F-5’- CGTGGCCTGATTCATTCC -3’
	lacZ-R-5’- ATCCTCTGCATGGTCAGGTC -3’
A8	WT (834 bp)
	F-5’-GCCTGCTTAGCCATCAGTCT-3’
	R-5’- GAAGGTCACTGAGGCTGGAA-3’
	KO (635 bp)
	F-5’-GCCTGCTTAGCCATCAGTCT-3’
	R-5’-GAAGGTCACTGAGGCTGGAA-3’
Ldlr$^{-/-}$	WT
	F-5’- ACC AGA CGT GCT CCC AGG ATG A -3’
	R-5’- CGC AGT GCT CCT CAT CGT ACT TGT C -3’
	KO
	F-5’- GAT TGG GAA GAC AAT AGC AGG CAT GC -3’
	R-5’- GGC AAG ATG GCT CAG CAA GCA AAG GC -3’
Lipg$^{-/-}$	WT
	F-5’-TTT TCC TCT TTC AAA GTG CTC CTG-3’
	R-5’-CCT ACG GGA ACT CCC TGA ACT TTT G -3’
	KO

	F-5'-TCA TTC TCA GTA TTG TTT TGC C -3'
	R-5'-AAG CCA GTG CTC ACC TAG C -3'
**Quantitative PCR**	
A3-(136-205)	F-5'-GCACCTTCAGAGCCAAAATCA-3'
	R-5'-GCAGGAGGCCATTCGCTAA-3'
A3-(2240-2314)	F-5'-GCATTCCCTAACTGCATATACAACA
	R-5'-ACCAGTCTTTCTGAGCTGATAAAAGAA
A3-(527-629)	F'-AGCAAGACAACAGCATAAGAGAACTC
	R'-CTGAGCTGCTTTTCTATTTCTTTTATCTG
A8-(434-547)	F-5'-CCCACCAAGAATTTGAGACCTT-3'
	R-5'-ACTGTTGCTGCTCTGCCATCT-3'
GAPDH-(230-231)	F-5'-TGTGTCCGTCGTGGATCTGA-3'
	R-5'-GGTGCCTCTGGAGATTTTCG-3'
18S	F-5'-AGGGTTCGATTCCGGAGAGG-3'
	R-5'-CAACTTTAATATACGCTATTGG-3'

Table S2. Offspring of Heterozygous Matings of *A3*$^{FL/FL}$ mice

	Males (n=77)				Females (n=69)					Total (n=146)			
	Observed		Expected		Observed		Expected			Observed		Expected	
	#	%	#	%	#	%	#	%		#	%	#	%
**(+/+)**	13	16.8	19.2	25	17	24.6	17.2	25	**Males**	77	55	73	50
**(+/-)**	44	57.1	38.5	50	34	49.3	34.5	50	**Females**	69	45	73	50
**(-/-)**	20	26.1	19.2	25	18	26.1	17.2	25					

Breedings			Pups		
Male	Female	No. of Litter	Pups per litter	Males	Females
(+/-)	(+/-)	33	4.4	77	69
(-/-)	(-/-)	51	3.6	96	86
(+/+)	(+/+)	37	4.3	90	71

Table S3. Offspring of Heterozygous Matings of *A3*$^{Nter/Nter}$ mice

	Males (n=39)				Females (n=60)					Total (n=99)			
	Observed		Expected		Observed		Expected			Observed		Expected	
	#	%	#	%	#	%	#	%		#	%	#	%
**(+/+)**	10	25.6	9.7	25	19	31.6	15	25	**Males**	39	39.3	49.5	50
**(+/-)**	18	46.2	19.5	50	25	41.6	30	50	**Females**	60	60.6	49.5	50
**(-/-)**	11	28.2	9.7	25	16	26.7	15	25					

Breedings			Pups		
Male	Female	No. of Litter	Pups per litter	Males	Females
(+/-)	(+/-)	19	5.2	39	60

Table S4. Offspring of Heterozygous Matings of *A8*$^{-/-}$ mice

	Males (n=79)				Females (n=62)					Total (n=141)			
	Observed		Expected		Observed		Expected			Observed		Expected	
	#	%	#	%	#	%	#	%		#	%	#	%
**(+/+)**	21	26.6	19.8	25	19	30.6	15.5	25	**Males**	79	56	70.5	50
**(+/-)**	39	49.3	39.5	50	31	50	31	50	**Females**	62	44	70.5	50
**(-/-)**	19	24.1	19.8	25	12	19.4	15.5	25					

Breedings			Pups		
Male	Female	No. of Litter	Pups per litter	Males	Females
(+/-)	(+/-)	34	4.2	79	62

Table S5. AlphaFold Structural Model Confidence

Enzyme	EL/LPL Residues	Inhibitor Complex	A3 Residues	A8 Residues	ipTM	pTM
NA	NA	A3-FL_3	17-460	NA	0.28	0.29
NA	NA	A3-FL_2A3-Nter	17-460; 17-221	NA	0.37	0.35
NA	NA	A3-$Nter_3$*	17-221	NA	0.54*	0.54
NA	NA	A3-$Nter_2$A8*	17-221	22-198	0.52*	0.52
NA	NA	A3-FL_2A8	17-460	22-198	0.39	0.39
EL	21-500	A3-FL_3	17-460	NA	0.27	0.35
EL	21-500	A3-FL_2A3-Nter*	17-460; 17-221	NA	0.37*	0.45
EL	21-500	A3-FL_2A8*	17-460	22-198	0.42*	0.46
EL	21-500	A3-FLA3-NterA8	17-460; 17-221	22-198	0.46	0.51
EL	21-500	A3-FLA3-$Nter_2$	17-460; 17-221	NA	0.42	0.47
EL	46-485	A3A8 hinge	35-152	27-143	0.49	0.68
EL	46-485	A3 hinge	35-152	NA	0.34	0.56
LPL	28-475	A3-FL_3	17-460	NA	0.28	0.36
LPL	28-475	A3-FL_2A3-Nter*	17-460; 17-221	NA	0.32*	0.39
LPL	28-475	A3-FL_2A8*	17-460	22-198	0.39*	0.45

*Complex structure models were generated by substituting A8 with A3-Nter, which is similar in size and domain content.

Table S6. Statistical analysis with exact P-values

**Fig2C**		
A3-FL: One-way ANOVA with Dunnett's multiple comparisons test	Summary	Adjusted P Value
+/+ vs. Full	+	1.69E-08
+/+ vs. Nter	**	6.02E-03
+/+ vs. -/-	**	3.93E-03
A3-Nter: One-way ANOVA with Dunnett's multiple comparisons test	Summary	Adjusted P Value
+/+ vs. Full	*	1.03E-02
+/+ vs. Nter	**	1.30E-03
+/+ vs. -/-	**	4.90E-03
A8: One-way ANOVA with Dunnett's multiple comparisons test	Summary	Adjusted P Value
+/+ vs. Full	*	4.69E-02
+/+ vs. Nter	ns	4.49E-01
+/+ vs. -/-	ns	1.08E-01
**Fig3A**		
One-way ANOVA with Tukey's multiple comparisons test	Summary	Adjusted P Value
+/+ vs. FL/FL	+	3.59E-07
+/+ vs. Nter/Nter	***	2.29E-04
+/+ vs. -/-	+	6.40E-11
FL/FL vs. Nter/Nter	***	9.63E-04
FL/FL vs. -/-	+	1.29E-06
Nter/Nter vs. -/-	+	2.39E-09
**Fig 3B**		
One-way ANOVA- Fisher's LSD		
+/+ vs FL/FL	**	1.15E-03
+/+ vs Nter/Nter	*	1.19E-02
FL/FL vs FL/Nter	+	7.19E-06
Nter/Nter vs FL/Nter	+	9.80E-05
**Fig 3C**		
One-way ANOVA with Tukey's multiple comparisons test	Summary	Adjusted P Value
+/+ vs. FL/FL	ns	9.93E-01
+/+ vs. Nter/Nter	*	2.40E-02
+/+ vs. -/-	+	1.30E-05
FL/FL vs. Nter/Nter	*	1.42E-02
FL/FL vs. -/-	+	8.62E-06
Nter/Nter vs. -/-	**	2.17E-03
**Fig 3E**		
One-way ANOVA- Fisher's LSD		

+/+: AAV-A8 vs GFP	+	1.01E-07
FL: AAV-A8 vs GFP	***	5.22E-04
Nter: AAV-A8 vs GFP	+	2.15E-06
+/+-AAV-A8 vs FL-AAV-A8	+	1.00E-05
+/+-AAV-A8 vs Nter-AAV-A8	ns	7.60E-02
FL-AAV-A8 vs Nter-AAV-A8	***	8.46E-04
**Fig 5B, left**		
Nonparametric Mann-Whitney test (unpaired, two tail)		
Ldlr$^{-/-}$ *vs Ldlr*$^{-/-}$*;A8*$^{-/-}$	*	2.80E-02
**Fig 5C**		
Unpaired two-tailed t test		
Panel: left	*	1.08E-02
Panel: right	**	3.53E-03
**Fig 5D_top panel**		
One-way ANOVA Tukey's multiple comparisons test		
Ldlr$^{-/-}$ *vs Ldlr*$^{-/-}$*;A8*$^{-/-}$	***	7.11E-04
Ldlr$^{-/-}$*;A8*$^{-/-}$ *vs Ldlr*$^{-/-}$*;A8*$^{-/-}$*;Lipg*$^{-/-}$	ns	7.15E-01
Ldlr$^{-/-}$*vs Ldlr*$^{-/-}$*;A8*$^{-/-}$*;Lipg*$^{-/-}$	***	7.82E-04
**Fig 5D_bottom panel**		
One-way ANOVA Tukey's multiple comparisons test		
Ldlr$^{-/-}$ *vs Ldlr*$^{-/-}$*;A8*$^{-/-}$	*	2.62E-02
Ldlr$^{-/-}$*;A8*$^{-/-}$ *vs Ldlr*$^{-/-}$*;A8*$^{-/-}$*;Lipg*$^{-/-}$	***	3.70E-04
Ldlr$^{-/-}$*vs Ldlr*$^{-/-}$*;A8*$^{-/-}$*;Lipg*$^{-/-}$	*	2.10E-02
**Fig 5F**		
One-way ANOVA- Fisher's LSD		
column 1 VS 3	**	8.17E-03
column 1 VS 5	+	1.90E-05
column 3 VS 4	+	2.80E-05
**Fig 5G**		
One-way ANOVA- Fisher's LSD	Summary	Individual P Value
Ldlr$^{-/-}$ vs. *Ldlr*$^{-/-}$*;A3*$^{-/-}$	+	5.19E-06
Ldlr$^{-/-}$ vs. *Ldlr*$^{-/-}$*;A8*$^{-/-}$	***	1.37E-04
Ldlr$^{-/-}$ vs. *Ldlr*$^{-/-;}$*A3*$^{-/-}$*;A8*$^{-/-}$	+	8.32E-06
Ldlr$^{-/-}$*;A3*$^{-/-}$ vs. *Ldlr*$^{-/-}$*;A8*$^{-/-}$	*	1.48E-02
Ldlr$^{-/-}$*;A3*$^{-/-}$ vs. *Ldlr*$^{-/-;}$*A3*$^{-/-}$*;A8*$^{-/-}$	ns	7.08E-01
Ldlr$^{-/-}$*;A8*$^{-/-}$ vs. *Ldlr*$^{-/-;}$*A3*$^{-/-}$*;A8*$^{-/-}$	*	3.13E-02
**Fig 6B**		
Paired two-tail t test		
12.5 ng, A3: A3+A8	**	3.00E-03
25 ng, A3: A3+A9	*	3.39E-02

**Fig 6D**		
**Left panel**		
One-way ANOVA- Fisher's LSD	Summary	Individual P Value
Column 1 vs. 2	**	9.10E-03
Column 1 vs. 4	*	4.67E-02
Column 2 vs. 3	**	1.10E-03
Column 2 vs. 4	ns	2.83E-01
Column 3 vs. 4	**	5.70E-03
**Right panel**		
One-way ANOVA- Fisher's LSD	Summary	Individual P Value
Column 2 vs. 3	***	1.00E-04
Column 2 vs. 4	**	1.46E-03
Column 3 vs. 4	**	2.60E-03
**Fig S2B**		
left, One-way ANOVA- Fisher's LSD	Summary	Individual P Value
+/+ vs. Nter	+	3.15E-07
FL vs. Nter	+	5.82E-07
Right, One-way ANOVA- Fisher's LSD	Summary	Individual P Value
+/+ vs. Nter	+	1.68E-09
FL vs. Nter	+	6.96E-10
**Fig S2C**		
One-way ANOVA- Fisher's LSD	Summary	Individual P Value
+/+ vs. FL/FL	+	2.53E-08
+/+ vs. Nter/Nter	*	4.93E-02
FL/FL vs. Nter/Nter	+	5.97E-09
**Fig S2E**		
Left: One-way ANOVA- Fisher's LSD	Summary	Individual P Value
WT vs. A3-Full	***	9.43E-04
WT vs. A3-N	*	1.75E-02
A3-Full vs. A3-N	+	4.50E-05
Right: Unpaired two-tailed t test		
A3-Full vs. A3-N	+	1.00E-05
**Fig S2F**		
One-way ANOVA- Fisher's LSD	Summary	Individual P Value
+/+ vs. FL	**	1.08E-03
FL vs. Nter	***	7.00E-04
**Fig S3B**		
Left: One-way ANOVA- Fisher's LSD	Summary	Individual P Value
WT vs. FL/-	*	1.28E-02
WT vs. FL/FL	****	4.69E-09
WT vs. FL/Nter	**	1.39E-03

FL/FL vs. FL/Nter	****	1.32E-06
Right: One-way ANOVA- Fisher's LSD	Summary	Individual P Value
WT vs. Nter/-	**	1.60E-03
Nter/- vs. Nter/Nter	**	5.70E-03
Nter/- vs. FL/Nter	**	2.50E-03
**Fig S5**		
Nonparametric Mann-Whitney test (unpaired, two tail)		
left: Ldlr$^{-/-}$ vs. Ldlr$^{-/-}$;A8$^{-/-}$	ns	5.00E-01
Right: Ldlr$^{-/-}$ vs. Ldlr$^{-/-}$;A8$^{-/-}$	*	2.32E-02
**Fig S6B**		
One-way ANOVA- Fisher's LSD		
Column 1 vs. 2	+	8.60E-09
Column 3 vs. 4	+	2.88E-10
**Fig S6C**		
One-way ANOVA- Fisher's LSD	Summary	Individual P Value
Ldlr$^{-/-}$ vs. *Ldlr*$^{-/-}$;*A3*$^{-/-}$	+	1.20E-05
Ldlr$^{-/-}$ vs. *Ldlr*$^{-/-}$;*A8*$^{-/-}$	+	1.00E-05
Ldlr$^{-/-}$ vs. *Ldlr*$^{-/-;}$*A3*$^{-/-}$;*A8*$^{-/-}$	+	1.50E-05
**Fig S6D**		
One-way ANOVA- Fisher's LSD	Summary	Adjusted P Value
**Triglyceride**		
WT vs. FL/FL	***	3.51E-04
WT vs. Nter/Nter	**	2.58E-03
FL/FL vs. Nter/Nter	ns	8.71E-02
**Cholesterol**		
WT vs. FL/FL	ns	2.80E-01
WT vs. Nter/Nter	**	3.96E-03
FL/FL vs. Nter/Nter	ns	1.28E-01

Figure S1

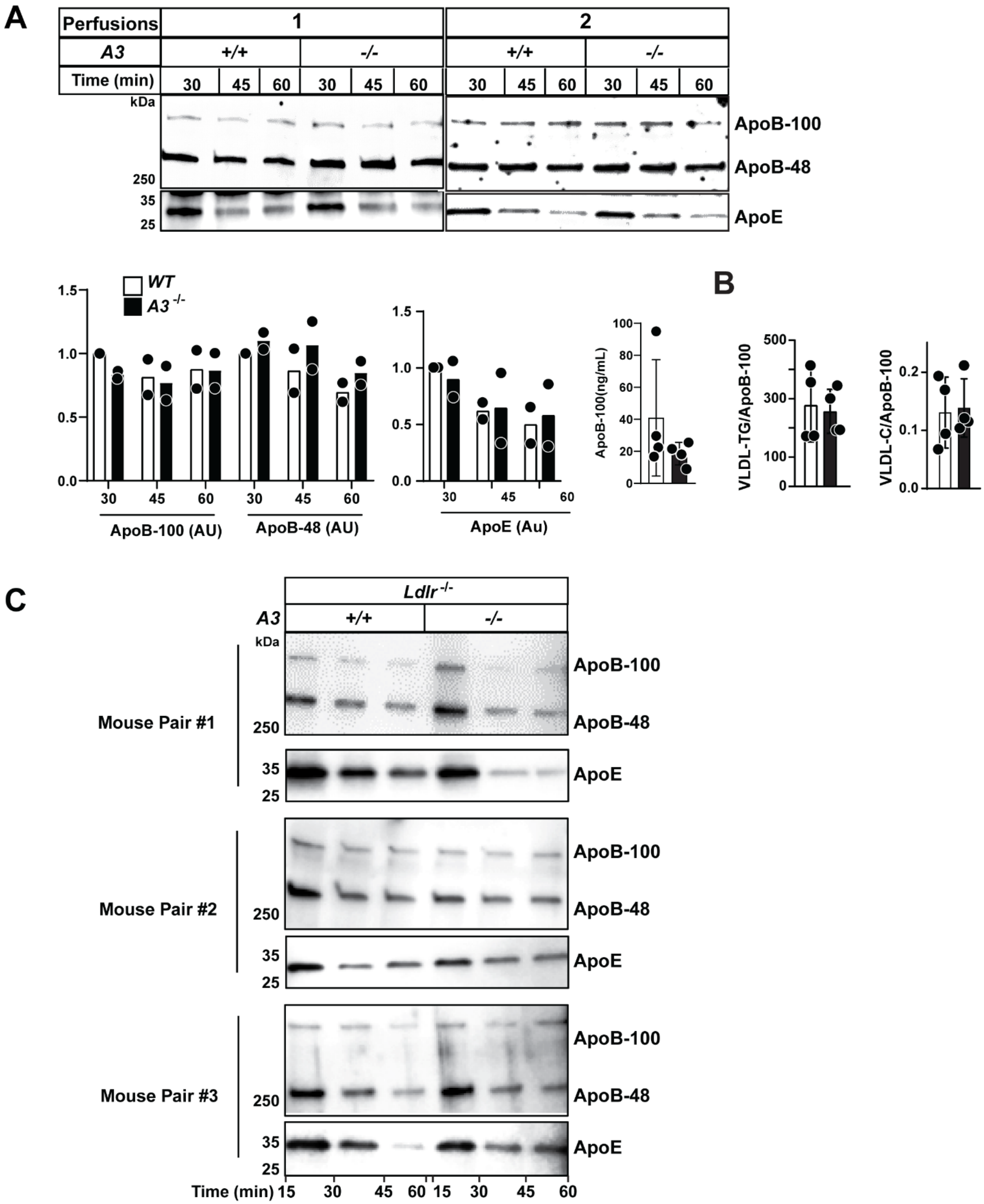
A
Perfusions
1
2
A3
+/+
-/-
Time (min)
30
45
60
kDa
250
35
25
ApoB-100
ApoB-48
ApoE
WT
A3 -/-
1.5
1.0
0.5
0.0
ApoB-100 (AU)
ApoB-48 (AU)
ApoE (Au)
ApoB-100(ng/mL)
B
VLDL-TG/ApoB-100
VLDL-C/ApoB-100
C
Ldlr -/-
Mouse Pair #1
Mouse Pair #2
Mouse Pair #3
Time (min) 15 30 45 60 15 30 45 60

Figure S1. Liver perfusion in WT and *A3*$^{-/-}$ mice without heparin treatment. A, Immunoblot analysis of ApoB and ApoE in isolated VLDL was performed as described in the Supplementary Methods (male, n=4/group, 10-12 wks, ad-lib fed). ApoB-100 levels were quantified by LI-COR(vales are normalized to the +/+ group at 30 min timepoint) and ELISA. **B,** Lipid content in VLDL measured enzymatically and normalized to ApoB-100 levels. **C**. Immunoblot of VLDL-ApoB and -ApoE in *Ldlr*$^{-/-}$;*A3*$^{-/-}$ and *Ldlr*$^{-/-}$ littermates (n=3/group, age matched 8-19 wks) after 2 wks on a high-sucrose diet. Each perfusion experiment included one WT and one *A3*$^{-/-}$ mouse. Mann-Whitney test was performed between WT and KO groups (A, right and B) (n=4), no significant differences were observed (p>0.05 for all comparisons). Four independent experiments were performed with similar results.

Figure S2

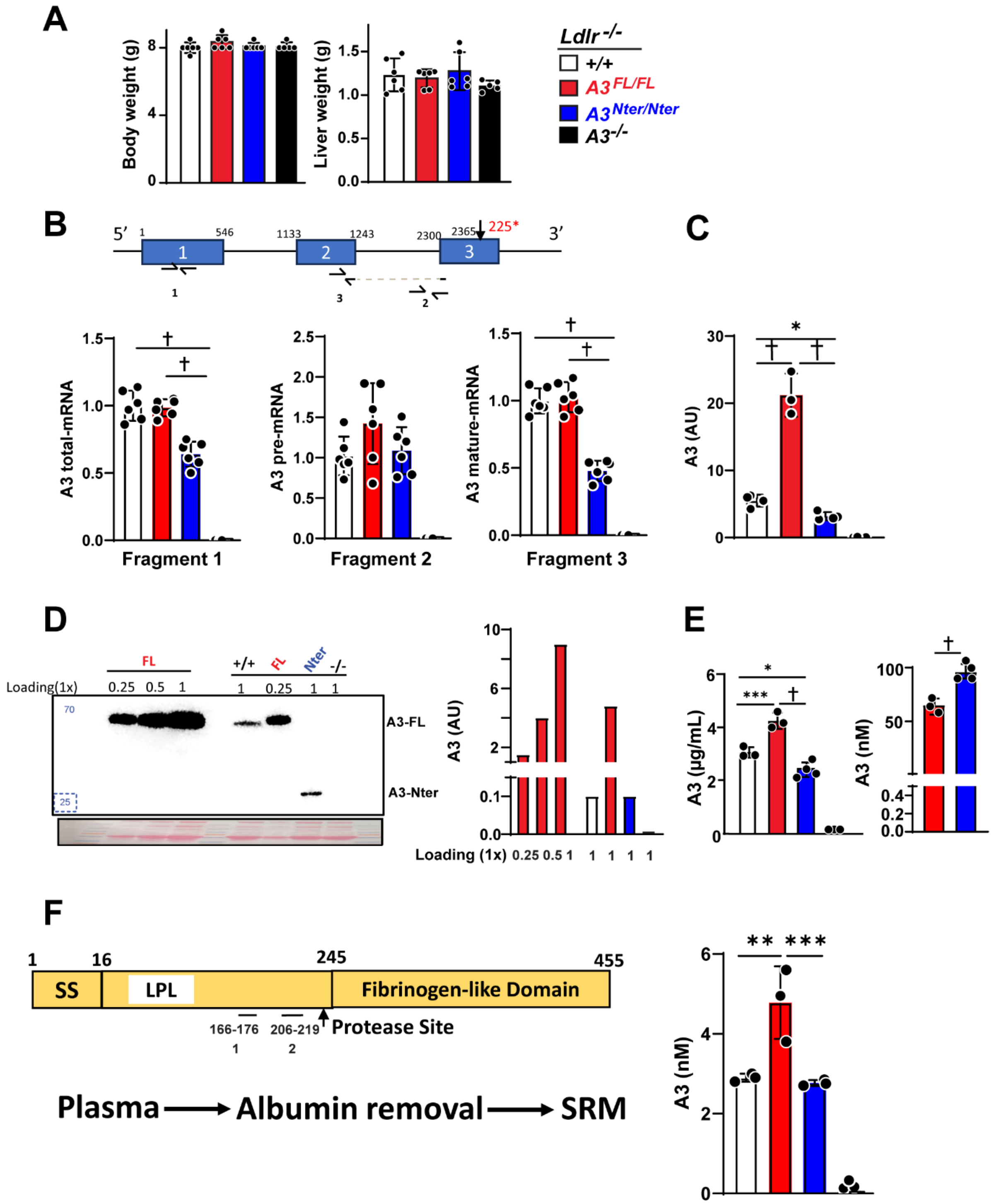

A
Body weight (g)
Liver weight (g)
Ldlr -/-
+/+
A3 FL/FL
A3 Nter/Nter
A3-/-
B
5′
3′
1
546
1133
1243
2300
2365
225*
Fragment 1
Fragment 2
Fragment 3
A3 total-mRNA
A3 pre-mRNA
A3 mature-mRNA
C
A3 (AU)
D
FL
+/+
FL
Nter
-/-
Loading(1x)
0.25 0.5 1
1 0.25 1 1
A3-FL
A3-Nter
A3 (AU)
Loading (1x) 0.25 0.5 1 1 1 1 1
E
A3 (µg/mL)
A3 (nM)
F
1
16
245
455
SS
LPL
Fibrinogen-like Domain
166-176
1
206-219
2
Protease Site
Plasma
Albumin removal
SRM
A3 (nM)

Figure S2. Effect of A3 cleavage on body weight, liver weight, mRNA and A3 protein levels. **A,** Body weights (left) and liver weights (right) of mice expressing A3 isoforms (male, n=6, 6, 6, 5/group, 11-16 wks of age, refed 4 h). **B**, Messenger RNA (mRNA) levels of A3 in indicated mouse strains. Top, Diagram of A3 with location of primers used for qRT-PCR. Bottom, qRT-PCR analysis of A3 mRNA levels in livers of refed male nice (8-12 wks old, n=6, 6, 6 and 5/group). Values are normalized to the +/+ group. **C**. Band intensities of A3-FL and A3-Nter were quantified by LI-COR in the indicated refed mice (males, 8-12 wks, n=4, 3, 4, 4/group). In the $A3^{FL/FL}$ mice, we included the signal at the expected molecular mass of the A3-Nter band; likewise, in the $A3^{Nter/Nter}$ mice, we included the signal at the expected molecular mass of A3-FL. Total A3 was calculated as the sum of A3-FL and A3-Nter in all mouse models. **D.** Immunoblot of plasma using an anti-A3-Nter mAb. Immunoblot analysis was performed using 1µL of plasma from the WT and A3-Nter mice and 0.25 µL from the A3-FL mice. Serial dilutions of plasma from the A3-FL were made using saline to establish the range in which the levels of detection were linear (left). **E**. Quantification of A3 in plasma by ELISA. Plasma was serially diluted to define the linear range of detection. Molarity of A3 in mice expressing A3-FL or A3-Nter was calculated from the mass measured by ELISA and the molecular weight of the peptides (65 and 27 kDa, respectively). Males, 7-9 wks, n=3, 3, 4, 3/group, refed 4 h. **F.** Quantification of A3 levels in plasma by SRM mass spectrometry. Left: Two peptides labeled with ^{13}C and ^{15}N, were used as standards to quantify the levels of A3 protein. Right: Circulating A3 levels were measured in plasma from refed male mice (8-12 wks, 3/group) after removal of albumin. The mean levels of A3 were determined by taking the mean of the three peptide measurements. Mean values were compared using nonparametric Kruskal-Wallis test followed by Dunn's procedure (A), parametric unpaired two-tailed student-t test (E, right) or one way ANOVA-Fisher's LSD test (B, E, F) in GraphPad Prism. All experiments

were repeated 3 times with similar results. Mean ± S.D. * $p < 0.05$ ** $p < 0.01$ *** $p < 0.001$ + $p < 0.0001$. Statistical values were not added to the graphs when no significant differences were found. When differences were statistically significant, the exact *p* values were provided in Supplementary Table 6**.**

Figure S3

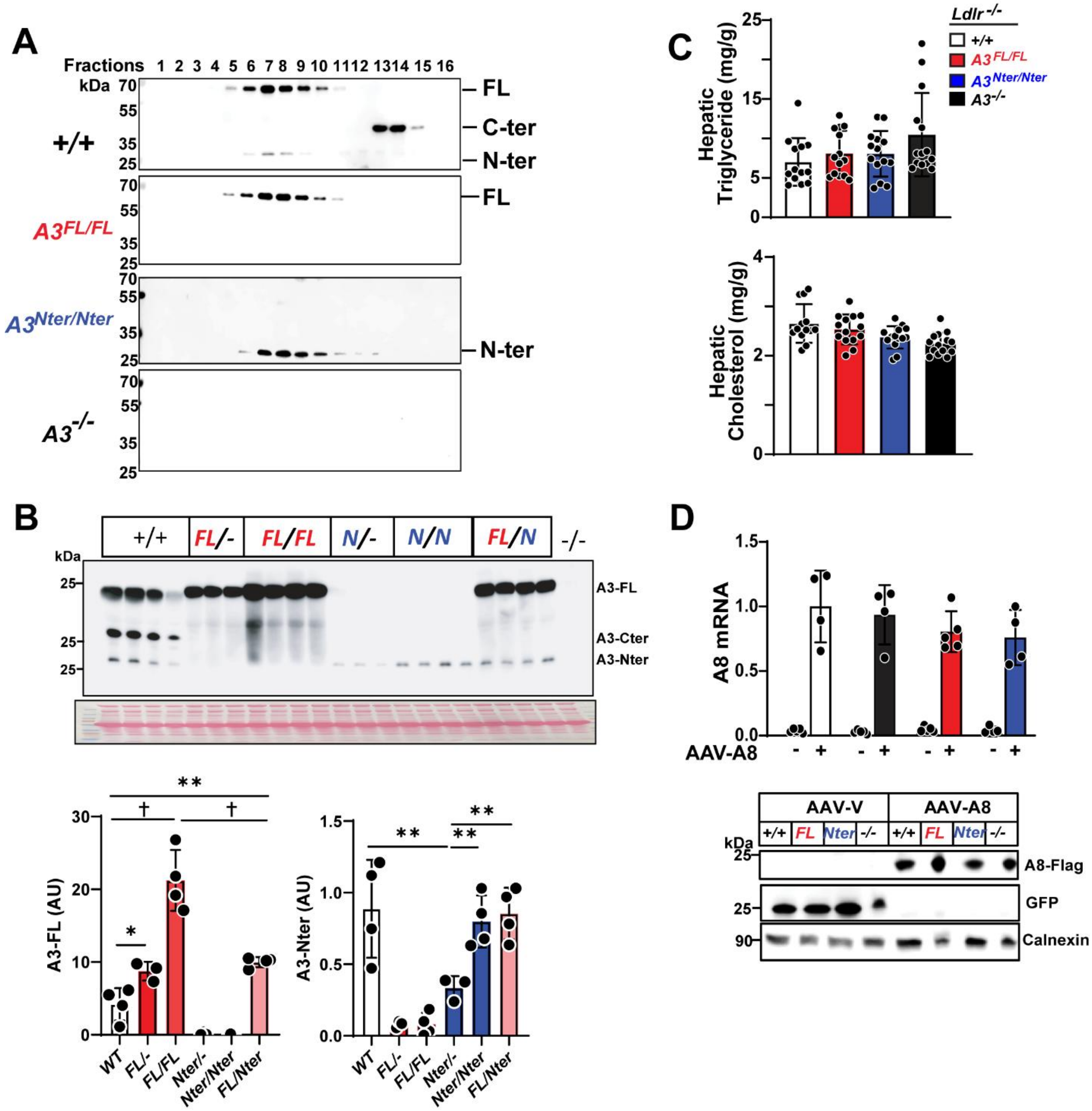

Figure S3. Effect of A3 cleavage on A3 hepatic lipid levels. A. Immunoblot of A3 in plasma fractions (12.5 µL per lane) separated by Superdex 200 size-exclusion chromatography (male, n=3, 3, 4, and 4/group, 8-12 wks, refed 4 h). **B**. Immunoblot analysis of plasma A3 in indicated mouse models. Assays were performed using plasma from refed age-matched male mice (n=4, 3, 4, 3, 4 and 4/group, 6-13 wks). Plasma was diluted 1:10 in saline and a total of 10 µl was loaded per lane.

Protein levels were quantified by LI-COR. **C**, Hepatic TG and cholesterol levels in 4 strains of mice. Data from 3 independent experiments were pooled (male, n=13, 14, 14, and 14/group, age matched 9-16 wks of age, refed 4 h). **D**, Hepatic *A8* mRNA and protein levels following A8 overexpression as described in Supplementary Methods (male, n=4, 4, 5, 4, 5, 5, 3, and 4/group, 8-12 wks, fast 16 h). Values are normalized to column 2 (+/+-AAV-A8 group). Mean values were compared using one-way ANOVA with Tukey' test (C) or one-way ANOVA with Fisher's LSD test (B, D). Mean ± S.D. * $p < 0.05$ ** $p < 0.01$ + $p < 0.0001$. Statistical values were not added to graphs when no significant difference was observed (p>0.05). When differences were statistically significant, the exact *p* values were provided in Supplementary Table 6. All experiments were repeated 3 times with similar results.

Figure S4

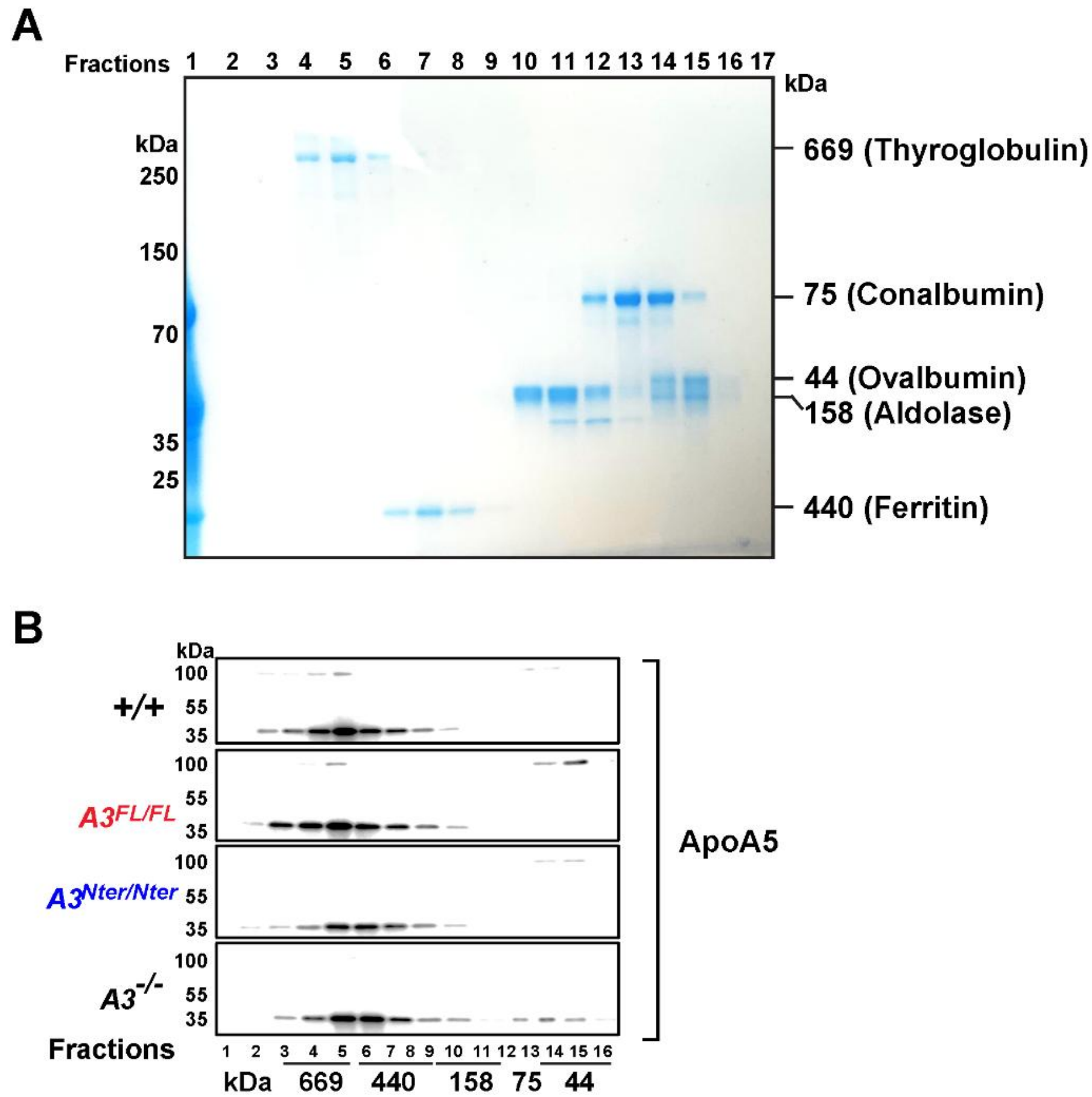

Figure S4. A3-FL and A3-Nter co-elute with ApoA5 following size-exclusion chromatography. A, Coomassie blue staining of molecular weight standards separated by a size exclusion column Superdex 200. A mixture of molecular weight standard makers was injected onto the column and fractionated using the same protocol as used for plasma samples. Odd-numbered fractions were collected, subjected to SDS-PAGE, and then stained with Coomassie blue. **B**, Immunoblot analysis of ApoA5 in plasma fractions separated by the size exclusion column Superdex 200 (male, n=6, 4, 6, and 6/group, 8-12 wks, refed 4 h). Experiments were repeated 3 times with similar results.

Figure S5

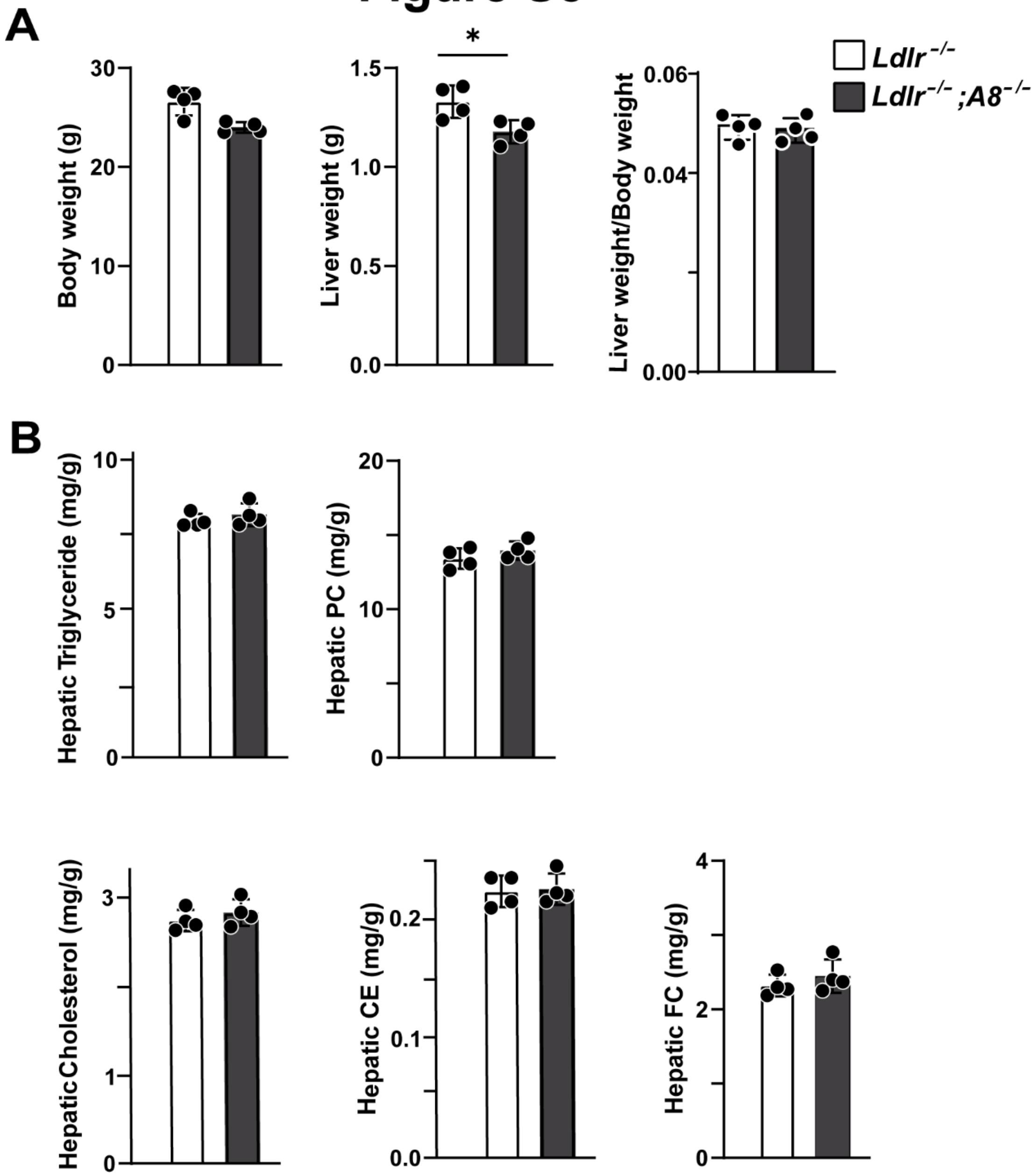

Figure S5. Effects of A8 inactivation on body, liver weight and hepatic lipid levels in *Ldlr*$^{-/-}$ mice. A, Body weight (left) and liver weight (right) in *Ldlr*$^{-/-}$ and *Ldlr*$^{-/-}$;A8$^{-/-}$ mice (male, n=4/group, 11-13 wks, refed 4 h). **p*=0.03. **B**, Hepatic lipid levels measured in the same strains of mice. Data is shown as mean ± SD. Statistical significance was determined using non-parametric Mann-Whitney test (unpaired, two-tailed) in GraphPad Prism. Statistical values were not added to

the graphs when no significant difference was observed ($p>0.05$). Experiments were repeated twice with similar results.

Figure S6

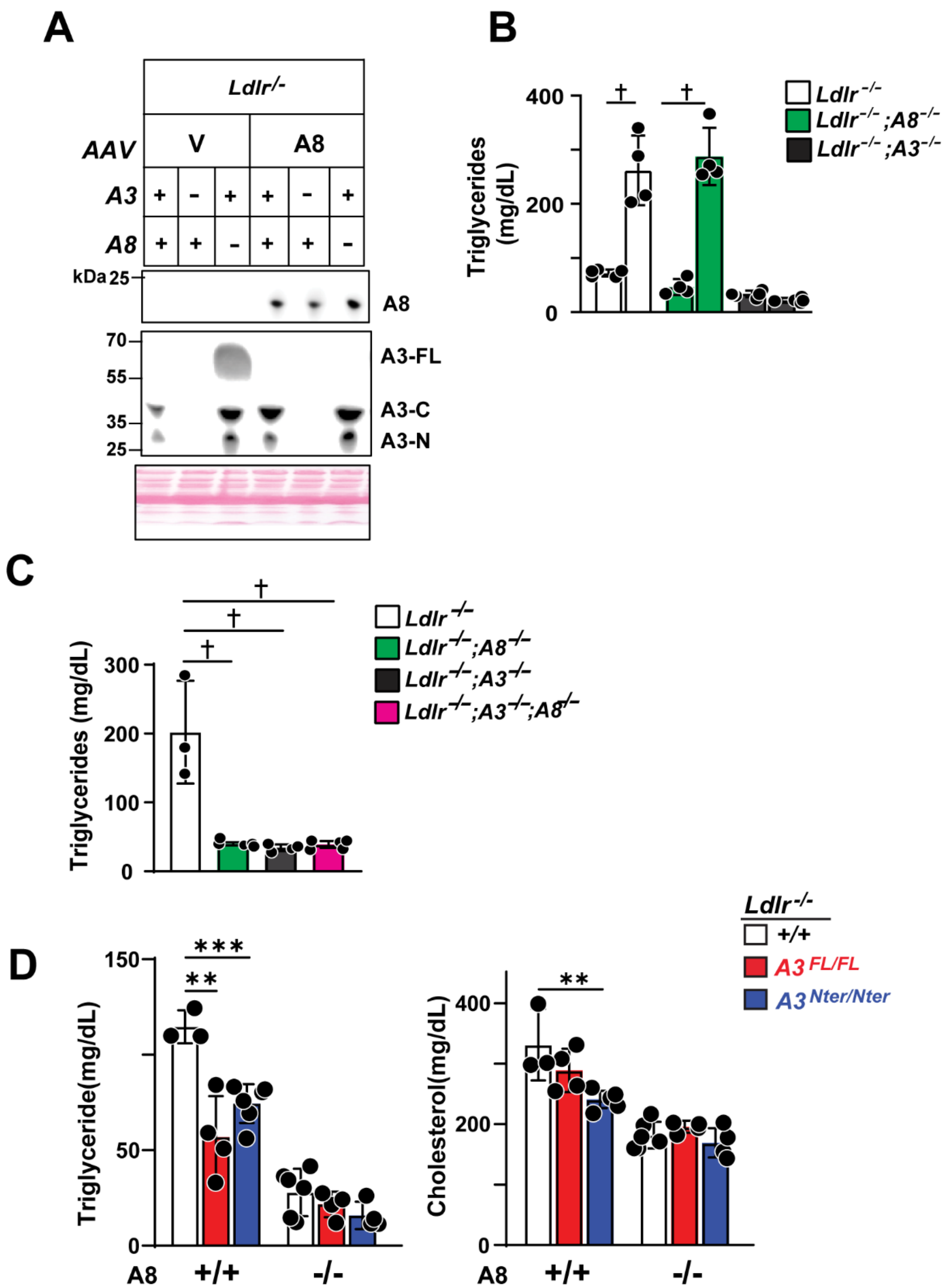

261

Figure S6. Both A3 and A8 are necessary to increase plasma levels of TG and cholesterol. A, Protein levels of circulating A8 and A3 and **B**, plasma TG levels following hepatic overexpression of A8 in *Ldlr*$^{-/-}$, *Ldlr*$^{-/-}$*;A8*$^{-/-}$, *Ldlr*$^{-/-}$*;A3*$^{-/-}$ mice, as described in Supplementary Methods and Figure 5F (male, n=5, 4, 4, 4, 5, and 5/group, 8-12 wks, fast 16 h). A FLAG antibody was used to detect A8, and AF136 pAb was used to detect A3 (10 μL per lane). **C**, Plasma TG levels in mice lacking both A3 and A8 (male, n=3, 5, 4, and 4/group, 8-11 wks, refed 4 h). **D,** Plasma TG and cholesterol levels in *Ldlr*$^{-/-}$, *Ldlr*$^{-/-}$*;A3*$^{FL/FL}$, *Ldlr*$^{-/-}$*;A3*$^{Nter/Nter}$ mice expressing or lacking *A8* (male, n=3, 4, 6, 6, 4, and 4/group, age matched 6-14 wks, refed 4 h). Mean values were compared using one -way ANOVA-Fisher LSD (B-D) in GraphPad Prism. Data are shown as mean ± SD. *p< 0.05, **p< 0.01, ***p<0.001, † p<0.0001. Exact p values were provided in Supplementary Table 6. Experiments were repeated twice with similar results.

Figure S7

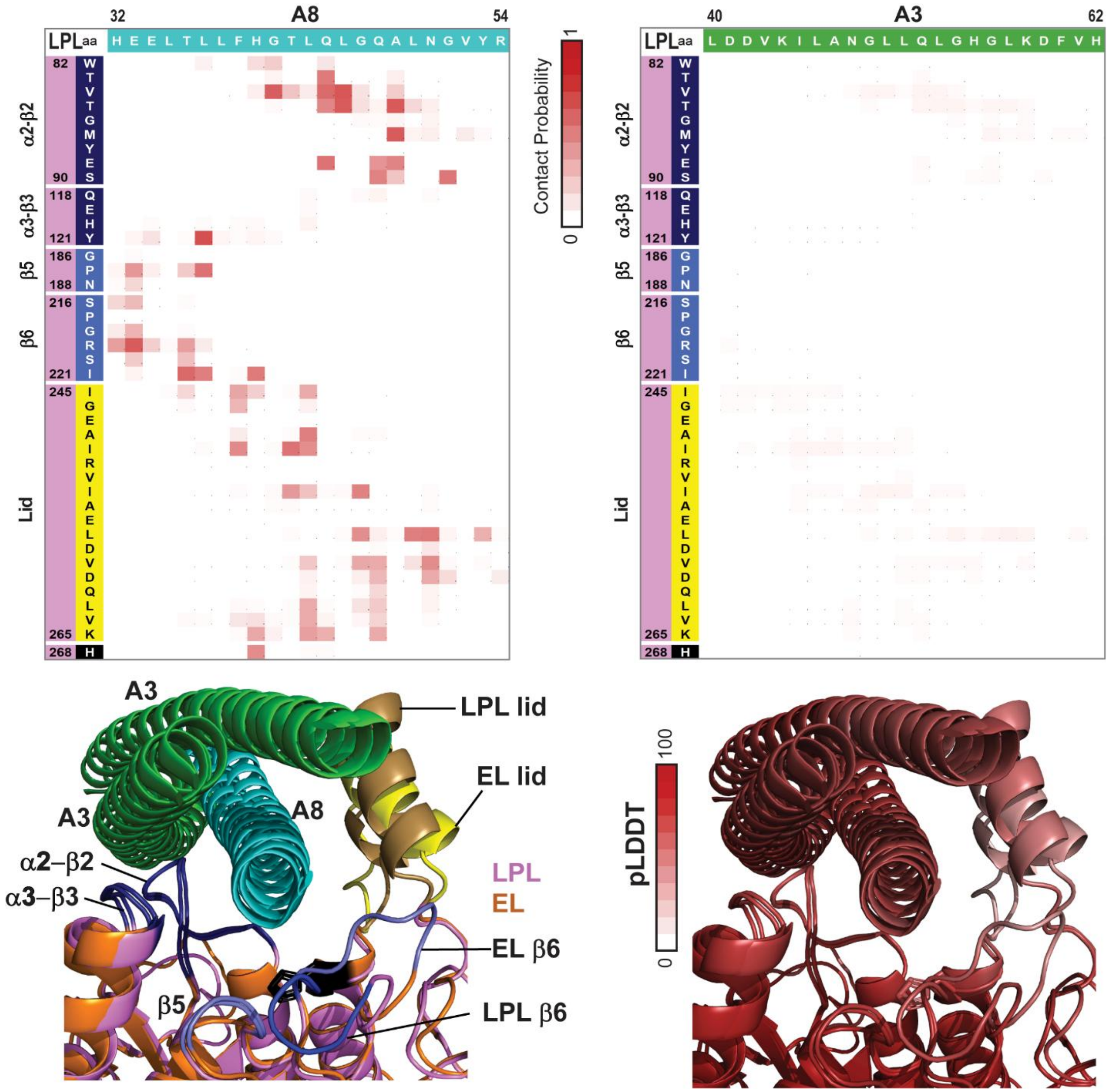

Figure S7. **Structural models of LPL: A3$_3$ and LPL: A3$_2$A8**. Top: Contact probabilities for residues in LPL with A8 (left) and A3 (right). Color scale bar: 0-1, units: contact probability. Bottom: superposition of EL and LPL complexes with A3$_2$A8 colored by structure element (left)

and complexes colored by pLDDT confidence values for AF models in white-red scale from 0 to 100 (right).

Supplemental References

53. Oldoni F, Cheng H, Banfi S, Gusarova V, Cohen JC, Hobbs HH. ANGPTL8 has both endocrine and autocrine effects on substrate utilization. *JCI Insight*. 2020. doi: 10.1172/jci.insight.138777

54. Burgess SC, Hausler N, Merritt M, Jeffrey FM, Storey C, Milde A, Koshy S, Lindner J, Magnuson MA, Malloy CR, et al. Impaired tricarboxylic acid cycle activity in mouse livers lacking cytosolic phosphoenolpyruvate carboxykinase. *J Biol Chem*. 2004;279:48941-48949. doi: 10.1074/jbc.M407120200

55. Luo F, Smagris E, Martin SA, Vale G, McDonald JG, Fletcher JA, Burgess SC, Hobbs HH, Cohen JC. Hepatic TM6SF2 is required for lipidation of VLDL in a Pre-Golgi compartment in mice and rats. *Cell Mol Gastroenterol Hepatol*. 2022;13:879-899. doi: 10.1016/j.jcmgh.2021.12.008

56. Engelking LJ, Kuriyama H, Hammer RE, Horton JD, Brown MS, Goldstein JL, Liang G. Overexpression of Insig-1 in the livers of transgenic mice inhibits SREBP processing and reduces insulin-stimulated lipogenesis. *J Clin Invest*. 2004;113:1168-1175. doi: 10.1172/JCI20978

57. Folch J, Lees M, Sloane Stanley GH. A simple method for the isolation and purification of total lipides from animal tissues. *J Biol Chem*. 1957;226:497-509. doi.org/10.1016/S0021-9258(18)64849-5

58. Jumper J, Evans R, Pritzel A, Green T, Figurnov M, Ronneberger O, Tunyasuvunakool K, Bates R, Zidek A, Potapenko A, et al. Highly accurate protein structure prediction with AlphaFold. *Nature*. 2021;596:583-589. doi: 10.1038/s41586-021-03819-2

59. Abramson J, Adler J, Dunger J, Evans R, Green T, Pritzel A, Ronneberger O, Willmore L, Ballard AJ, Bambrick J, et al. Accurate structure prediction of biomolecular interactions with AlphaFold 3. *Nature*. 2024;630:493-500. doi: 10.1038/s41586-024-07487-w

60. Varga JK, Ovchinnikov S, Schueler-Furman O. actifpTM: a refined confidence metric of AlphaFold2 predictions involving flexible regions. *Bioinformatics*. 2025;41. doi: 10.1093/bioinformatics/btaf107
